# Effects of broad-spectrum antibiotics on the colonisation of probiotic yeast *Saccharomyces boulardii* in the murine gastrointestinal tract

**DOI:** 10.1038/s41598-022-12806-0

**Published:** 2022-05-25

**Authors:** Karl Alex Hedin, Vanessa Emily Rees, Hongbin Zhang, Vibeke Kruse, Ruben Vazquez-Uribe, Morten Otto Alexander Sommer

**Affiliations:** Novo Nordisk Foundation Center for Biosustainability, Technical University of Denmark, 2800 Kgs. Lyngby, Denmark

**Keywords:** Microbiology, Gastrointestinal models, Model fungi, Biological techniques, Mouse, Molecular engineering

## Abstract

Mouse models are commonly used to study the colonisation profiles of microorganisms introduced to the gastrointestinal tract. Three commonly used mouse models include conventional, germ-free, and antibiotic-treated mice. However, colonisation resistance in conventional mice and specialised equipment for germ-free mice are usually limiting factors in their applications. In this study, we sought to establish a robust colonisation model for *Saccharomyces boulardii*, a probiotic yeast that has caught attention in the field of probiotics and advanced microbiome therapeutics. We characterised the colonisation of *S. boulardii* in conventional mice and mice treated with a cocktail of broad-spectrum antibiotics, including ampicillin, kanamycin, metronidazole and vancomycin. We found colonisation levels increased up to 10,000-fold in the antibiotic-treated mice compared to nonantibiotic-treated mice. Furthermore, *S. boulardii* was detected continuously in more than 75% of mice for 10 days after the last administration in antibiotic-treated mice, in contrast to in nonantibiotic-treated mice where *S. boulardii* was undetectable in less than 2 days. Finally, we demonstrated that this antibiotic cocktail can be used in two commonly used mouse strains, C57BL/6 and *ob/ob* mice, both achieving ~ 10^8^ CFU/g of *S. boulardii* in faeces. These findings highlight that the antibiotic cocktail used in this study is an advantageous tool to study *S. boulardii* based probiotic and advanced microbiome therapeutics*.*

## Introduction

The human body hosts trillions of microorganisms, consisting of a diverse range of different communities of commensal, pathogenic and symbiotic microorganisms that together make up the human microbiota^1,2^. These communities reside in numerous environmental sites on and within the human body, such as the skin, oral cavity, respiratory tract and gastrointestinal tract^3^. The gastrointestinal tract contains the most abundant and diverse microbial communities in the human body^2^. The attributes of these communities in the gastrointestinal tract play an essential role in sensing, responding and manipulating the human gut environment^4^. Recent studies on the function of the human gut microbiota support it as a potential target to prevent numerous diseases, including diabetes, cancer, mental health, and obesity^5–10^ via probiotic administration or faecal matter transplants^11–13^.

In addition, an emerging therapeutic modality involves engineered probiotic microorganisms to create advance microbial therapeutics (AMTs)^14^. These engineered microorganisms may exhibit therapeutic activities in the gastrointestinal tract, skin, or blood of their hosts^15–19^. Commonly used probiotic bacteria have been utilised as AMT chassis; for example, *Lactococcus lactis* and *Lactobacillus gasseri* have been engineered to prevent exacerbation of diabetes^20–23^, and *Escherichia coli* Nissle 1917 to treat phenylketonuria^16^ and hyperammonaemia^17^. Bacteria offer some benefits as AMT chassis due to their simplicity of engineering and as they natively occur in high abundance in the gut^2^. However, for the expression of peptides with post-translational modifications of proteins, eukaryotic hosts may be advantageous.

The probiotic yeast, *Saccharomyces cerevisiae* var. *boulardii*, a subspecies of the commonly used baker’s yeast, *Saccharomyces cerevisiae,* has caught the attention within the field of probiotics and AMTs^24–27^. *S. boulardii* taken as a probiotic supplement has been reported to ameliorate certain antibiotic-associated disorders^28^, such as *Clostridium difficile* infections^29,30^, inflammatory bowel disease^31^ and other gastrointestinal disorders^28,32^. Established genetic tools for *S. cerevisiae*^26,33,34^ can also be applied to *S. boulardii* as they are closely related^35,36^, allowing *S. boulardii* to be used as an AMT chassis. Examples of this include engineering *S. boulardii* to secrete IL-10 to enhance its anti-inflammatory properties^37^ and to secrete atrial natriuretic peptide to treat murine colitis^38^, thus demonstrating *S. boulardii* as a promising AMT platform, in addition to a probiotic itself.

Regrettably, *S. boulardii* has not been considered an efficient coloniser in conventional rodents with an intact microbiome due to its low abundance and short residence time in the gastrointestinal tract^25,26,39,40^. For instance, in conventional mice *S. boulardii* was completely washed-out after two days, even though it can survive the low pH of the stomach and harsh conditions of gastrointestinal tract^41,42^. Conversely, *S. boulardii* administered to germ-free mice colonise the gastrointestinal tract for 30 days after a single administration^26^. However, the cost and accessibility of such mouse model are limiting factors. Studying a disease that requires a stable delivery of the therapeutic would require more consistent *S. boulardii* levels. However, accessibility to a germ-free murine model and poor colonisation in conventional murine models complicates the preclinical assessment of the probiotic yeast. Considering the importance of robustness in preclinical assessment, a need remains for standardised colonisation and testing of *S. boulardii* in murine models. To address this problem, we sought to establish an antibiotic-treated model for *S. boulardii* to achieve reproducible and robust colonisation in mice.

## Results

### Pre-treatment with an antibiotic cocktail significantly extends *Saccharomyces boulardii* colonisation in mice

Use of an antibiotic-treated mice model for *S. boulardii* should reduce the potential variability in colonisation introduced with the environment, diet, breed, and other factors, which alter the gut microbiota^43^. We hypothesised that treatment with a combination of broad-spectrum antibiotics would eliminate gut bacteria competing with *S. boulardii,* allowing for more robust colonisation and better reproducibility. We used an antibiotic cocktail containing ampicillin, kanamycin, metronidazole, and vancomycin enabling us to target multiple bacteria in the murine gastrointestinal tract^44–46^. We evaluated the in vitro growth performance of *S. boulardii* in the presence of each antibiotic individually and in combination (Supplementary Fig. S1).

**Figure 1:**
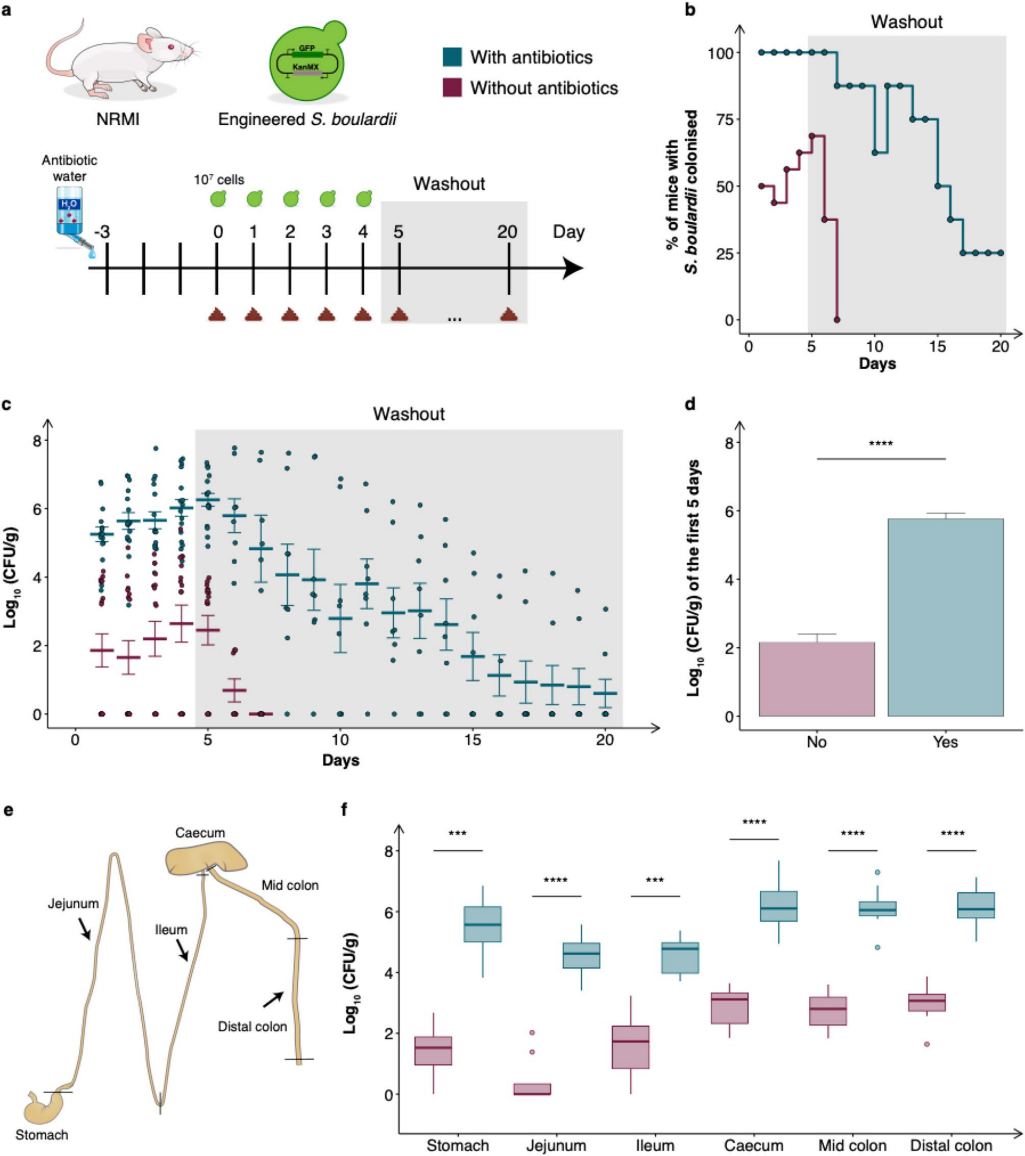
Colonisation assessment of *S. boulardii* in antibiotic-treated mice. (**a**) A graphical scheme of the study design. The mice were orally administered ~ 10^7^ cells of *S. boulardii* daily for five successive days, followed by a 15-day washout period (grey shaded area). The mice were divided into two cohorts, where one received antibiotics supplemented in the drinking water. On the fifth day, half of the mice were sacrificed to determine the colonisation of *S. boulardii* in different gut regions. (**b**) The step plot shows the time-course of percentage of mice with detectable levels of *S. boulardii* in the antibiotic-treated cohort (blue) and the none-antibiotic-treated cohort (red). (**c**) The time-course of the log_10_ mean CFU/g of *S. boulardii* ± SEM mean over time (days) in faeces. Each dot represents the log_10_ CFU/g *S. boulardii* in one mouse. (**d**) Bar plot of the mean CFU/g of *S. boulardii* assessed in the faeces over the first five days. (**e**) A graphical illustration of the different gut regions investigated and how the regions were defined as marked. (**f**) Box plot of the assessed CFU/g of *S. boulardii* in the gastrointestinal tract from the nonantibiotic-treated and antibiotic-treated mice sacrificed on the fifth day. *p < 0.5, **p < 0.1, ***p < 0.01, ****p < 0.001. All samples were analysed by a dependent sample t-test.

We evaluated the effect on colonisation and washout of *S. boulardii* in two cohorts of mice receiving either drinking water supplemented with antibiotics or normal drinking water (Fig. 1a). To confidently quantify colonisation levels, we orally administered ~ 10^7^ cells of a genetically engineered *S. boulardii* expressing green fluorescent protein (GFP) and an antibiotic resistance selection marker (geneticin selectable marker, KanMX). Antibiotic supplemented drinking water enhanced the colonisation of *S. boulardii* with 100% of the treated mice showing detectable colonisation of *S. boulardii* for the first six days, with 75% remaining with detectable levels for a prolonged period up to 10 days (Fig. 1b). In contrast to the nonantibiotic-treated mice that demonstrated complete washout of *S. boulardii* after a short period (up to 2 days). Additionally, we observed a more than a 1,000-fold increase of viable CFU/g in faeces during the period of daily oral administrations of *S. boulardii* for the antibiotic-treated mice compared to nonantibiotic-treated mice (Fig. 1c–d).

We further determined the colonisation of *S. boulardii* in different regions of the gastrointestinal tract. In all of the collected gut regions we observed a significant increase of viable *S. boulardii* CFU in the antibiotic-treated mice (Fig. 1e–f). Furthermore, *S. boulardii* was able to colonise all regions of the antibiotic-treated mice, while in the nonantibiotic-treated mice it was undetectable for most of the stomach and small intestine samples (Fig. 1f).

### Antibiotic cocktail opens a niche in the murine gastrointestinal tract for *S. boulardii* colonisation

**Figure 2:**
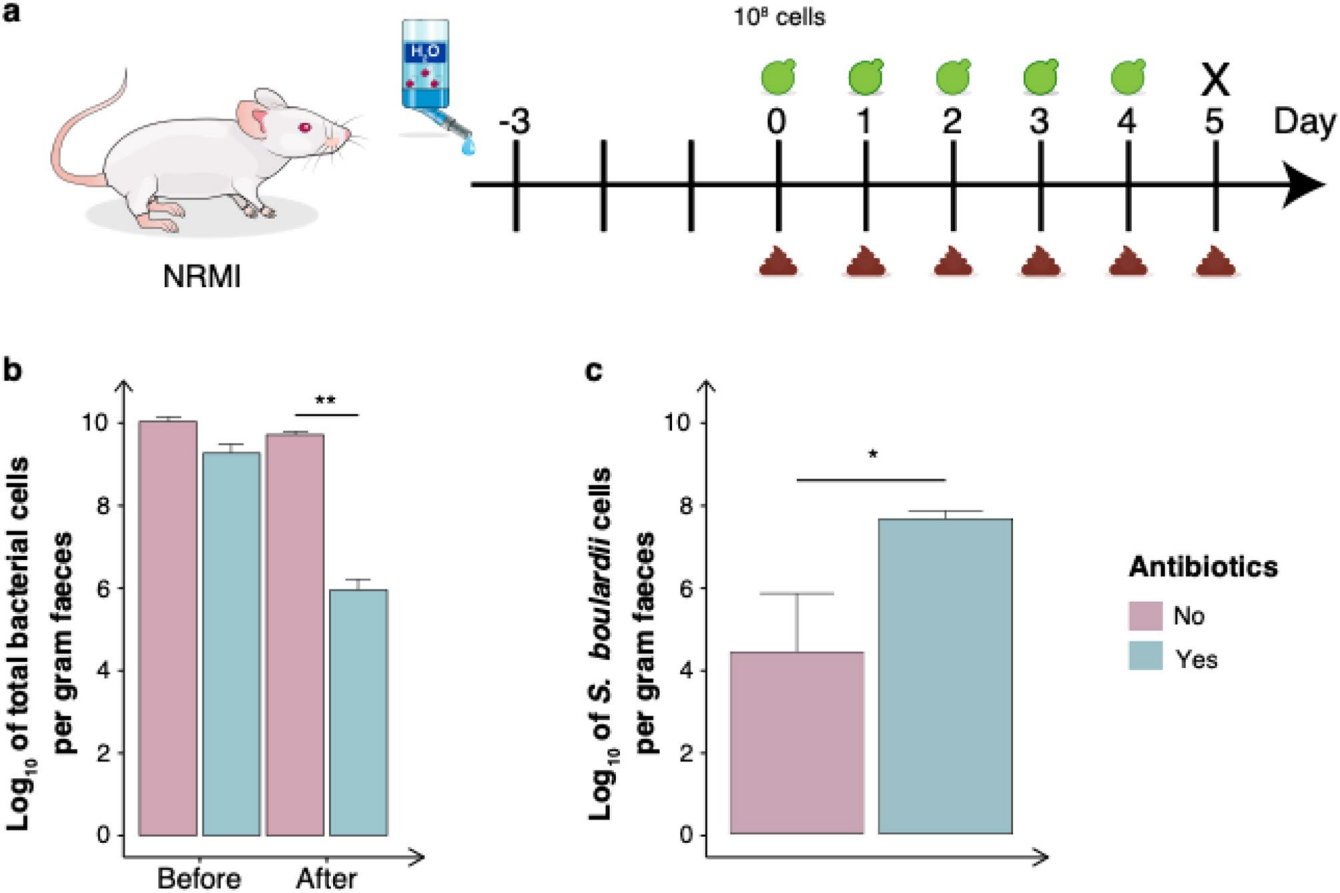
The effect of *S. boulardii* on the bacterial communities in antibiotic-treated mice. (**a**) Graphical scheme of the study design. The mice were orally administered ~ 10^8^ cells of *S. boulardii* daily for five successive days. The mice were divided into two cohorts, where one received antibiotics supplemented in the drinking water. (**b**) Log_10_ number of total bacterial cells per gram of faeces before and after receiving the antibiotics. (**c**) Bar plot of the mean CFU/g assessed in faeces over the first five days. Bar plots are shown as the mean ± SEM *p < 0.5, **p < 0.1, ***p < 0.01, ****p < 0.001. All samples were analysed by Wilcoxon signed-rank test.

Once the residence time was assessed we sought to investigate the effect on the total bacterial communities in antibiotic-treated mice. The mice were orally administered ~ 10^8^ cells of *S. boulardii* and faecal samples were collected daily until sacrifice on day five (Fig. 2a). Receiving an antibiotic cocktail reduced the total bacterial abundance by four orders of magnitude (Fig. 2b). Additionally, receiving the oral administration of ~ 10^8^ *S. boulardii* cells together with the antibiotic cocktail resulted in an abundance three orders of magnitude greater than the nonantibiotic-treated mice (Fig. 2c).

### Receiving an antibiotic cocktail enables *Saccharomyces boulardii* to achieve reproducible and robust colonisation in two common mouse strains

**Figure 3:**
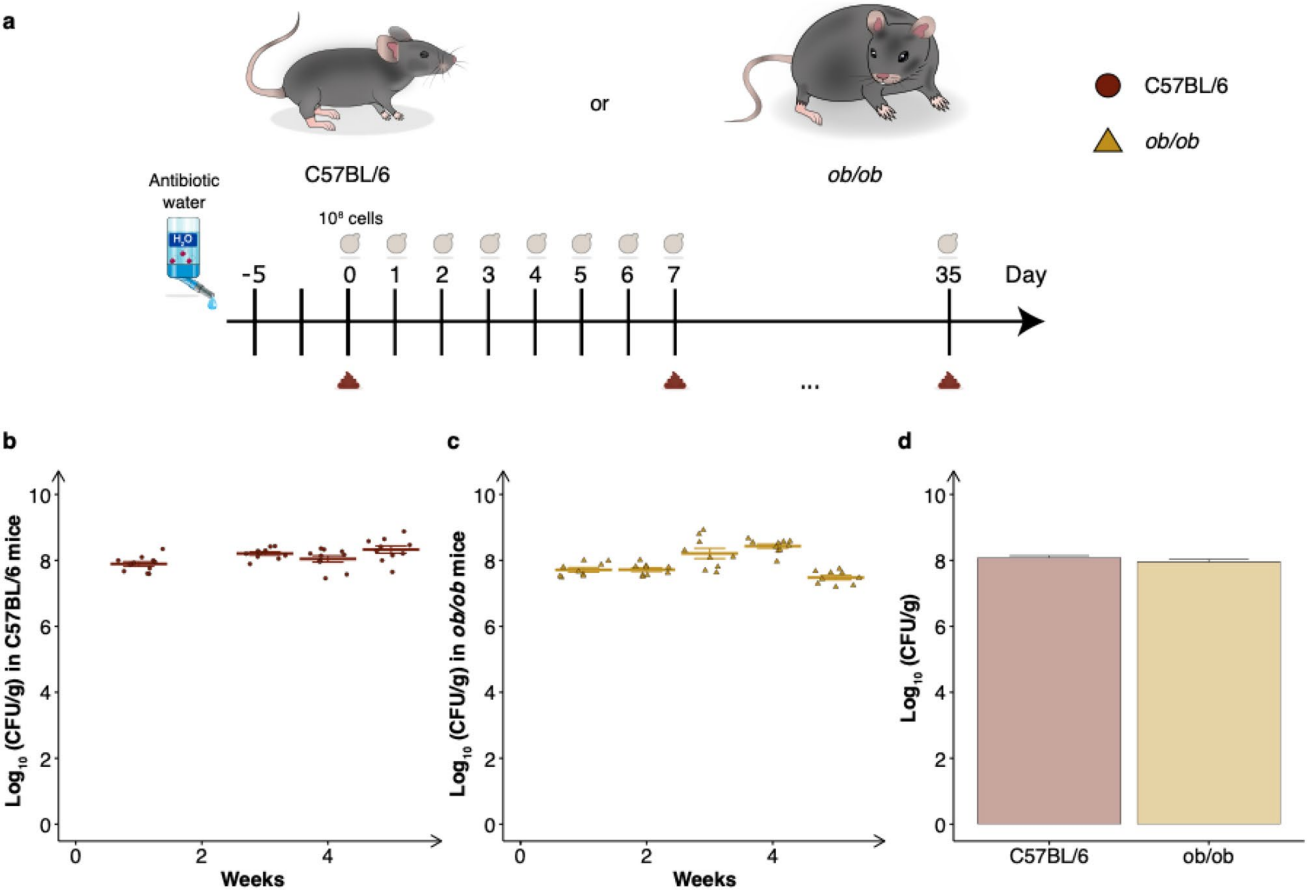
Colonisation assessment of *S. boulardii* in two different mouse strains receiving antibiotic-treatment. (**a**) A graphical scheme of the study design. The mice were orally administered ~ 10^8^ cells of *S. boulardii* daily for five successive weeks, with weekly faecal collection. (**b**) The time-course of log_10_ mean CFU/g of *S. boulardii* ± SEM over time (weeks) in faeces from C57BL/6 mice. (**c**) The time-course of log_10_ mean CFU/g of *S. boulardii* ± SEM over time (weeks) in faeces from *ob/ob* mice. (**d**) Bar plot of the mean CFU/g assessed in the faeces over the same period. Each dot represents the log_10_ CFU/g *S. boulardii* in one mouse.

After establishing the residence time of *S. boulardii* following its administration and assessing the effect of antibiotics on the colonisation of *S. boulardii*, we sought to investigate the robustness with the antibiotic cocktail in two different mouse strains. We chose two of the most commonly used mouse models, the C57BL/6 fed with a high fat diet and the leptin deficient *ob/ob* mouse strain*.* The C57BL/6 mouse strain is used frequently in research including that for metabolic diseases, oncology, immunology and toxicology^47^, whilst the *ob/ob* strain is commonly used for studying Type II diabetes and obesity^48,49^. These colonisation studies were performed independently, with oral administration of probiotic *S. boulardii* from two different batches that were delivered daily for five weeks (Fig. 3a). Notably the abundance in faeces reached ~ 10^8^ CFU/g in both murine strains and maintained at a high abundance (~ 10^8^ CFU/g) throughout the five weeks (Fig. 3b–d).

## Discussion

Since the early days of microbiome research, mouse models have been used to investigate how microbes interact with their hosts. In particular, germ-free and antibiotic-treated mice have been a valuable tool to study the direct interaction of a single or a defined community of microbes in the gastrointestinal tract^45^. In this study, we characterised the effect of an antibiotic cocktail to build a robust mouse model for colonisation of *S. boulardii*. We started with orally administered ~ 10^7^ cells (Fig. 1a) to correlate our observed colonisation with previous studies with *S. boulardii*^26^. The oral administered *S. boulardii* was later increased by tenfold to boost to colonisation (Figs. 2a, 3a). Here we demonstrated that treating mice with a cocktail of antibiotics enabled *S. boulardii* to remain in 75% of mice for 10 days and 25% of mice for 15 days (Fig. 1b) following the last oral administration. This was in stark contrast to the colonisation observed in the nonantibiotic-treated mice that demonstrated complete washout of *S. boulardii* after two days. This latter observation appears identical to a previous study that compared *S. boulardii* in mice treated with a cocktail of penicillin (1 mg/mL) and streptomycin (2 mg/mL), where *S. boulardii* still appeared to washout after two days^26^. This emphasised the importance of the use of the broad-spectrum antibiotic cocktail in this study. In addition, we observed more than a 1,000-fold increase in viable count of *S. boulardii* in faeces from antibiotic-treated mice compared to nonantibiotic-treated mice (Figs. 1d, 2c).

The gut microbiota of mice from the same breed has previously been shown to differ significantly between suppliers resulting in challenges to reproduce experiments^50–52^. In this study, we demonstrated the robustness of the antibiotic cocktail model, showing an insignificant difference in colonisation for the first four weeks between two different studies (Fig. 3b–d). We showed that the antibiotic cocktail in mice exhibits reproducible colonisation of *S. boulardii* in two different mouse strains (C57BL/6 and *ob/ob*) under two different diets (standard chow and high fat diet). These studies were conducted independently with two different batches of *S. boulardii*, maintaining ~ 10^8^ CFU/g in faeces for up to five weeks (Fig. 3b). Interestingly, here we observed ~ tenfold higher CFU/g counts in faeces of antibiotic-treated mice compared to previously reported numbers in germ-free mice^26^.

Comparing the viable counts in the different regions, we observed a significant increase in all regions by supplementing antibiotics in the drinking water, highlighting the potential of *S. boulardii* to act on multiple sections of the gastrointestinal tract (Fig. 1f). We observed the largest increase in viable cells in the jejunum and stomach, which increased by ~ 10,000-fold when antibiotics were supplemented. This was followed by the ileum and lower intestinal tract, which all increased by ~ 1,000-fold viable cells. Furthermore, the ability of *S. boulardii* to colonise the antibiotic-treated mice were 10- to 100-fold lower than some of the more common bacterial probiotics^53,54^, such as *E. coli* Nissle 1917 which can reach up to 10^9^ cells/g in faeces^55^.

Treating mice with broad-spectrum antibiotics has been commonly used to deplete bacterial populations in the gastrointestinal tract. We used a cocktail of antibiotics that has previously been demonstrated to open a niche in the gastrointestinal tract^44–46^. In our study, using this antibiotic cocktail showed no growth burden for *S. boulardii* (Supplementary Fig. S1). Distinctly, the antibiotic cocktail of this study significantly impacted the total bacterial abundance, as observed by a 10,000-fold decrease of bacterial cells (Fig. 2b). Although the treatment cannot completely eliminate the gut microbiota^56^, it represents a rapid, inexpensive and accessible alternative to the germ-free model^45^. Further studies would be necessary to fully elucidate the long-term colonisation effects and dynamics of *S. boulardii* as well as other fungal species on the host. Indeed, it has been demonstrated that antibiotics have a long-term effect on the fungal community in the gut, shifting from mutualism towards competition^57^. To reduce the risk of other fungal species taking over or competing with *S. boulardii,* anti-fungal compounds that *S. boulardii* has been engineered to resist could be added to the antibiotic cocktail.

Investigation of physiological effects of *S. boulardii* based probiotics and AMTs requires animal models that allow robust and consistent colonisation. In this study, we demonstrated that treatment with an antibiotic cocktail led to reproducible and robust colonisation of *S. boulardii* throughout the gastrointestinal tract. This model may be useful as a rapid, low-cost setup for testing *S. boulardii* AMTs and probiotics.

## Methods

### Strains and media

One Shot® TOP10 *Escherichia coli* (Thermo Fisher Scientific, Waltham, MA, USA) were used for plasmid construction and maintenance. *E. coli* cultures were grown in lysogeny broth (LB) media containing 5 g/L yeast extract, 10 g/L tryptone and 10 g/L NaCl; (Sigma Aldrich—Merck Life Science) supplemented with 100 mg/L ampicillin sodium salt (Sigma Aldrich—Merck Life Science). *S. boulardii* (*S. cerevisiae* ATCC® MYA796^TM^) was obtained from American Type Culture Collection, ATCC. The strains created in this study are listed in Supplementary Table S2. *S. boulardii* were cultured in yeast extract peptone dextrose (YPD) media containing 10 g/L yeast extract, 20 g/L casein peptone and 20 g/L glucose; (Sigma Aldrich—Merck Life Science) at 37 °C. Strain selection was done on YPD plates containing 20 g/L agar and supplemented with 200 mg/L geneticin (G418; Sigma Aldrich—Merck Life Science) or synthetic complete (SC) plates containing 1.7 g/L yeast nitrogen base without amino acids and ammonium sulphate (Sigma Aldrich—Merck Life Science), 1 g/L monosodium glutamate (Sigma Aldrich—Merck Life Science), 1.92 g/L Yeast Synthetic Drop-out Medium Supplements without uracil (Sigma Aldrich—Merck Life Science) and 0.2 g/L uracil (Sigma Aldrich—Merck Life Science) at 37 °C.

### Strain and plasmid construction

The oligonucleotides and gBlock sequences were codon-optimised and ordered from Integrated DNA Technologies (IDT; sequences are listed in Supplementary Information Table S1). All plasmids used and generated in this study are listed in Supplementary Information Table S3. Phusion high-fidelity DNA polymerase (Thermo Scientific, Waltham, MA, USA) was used for amplifying GFP. GFP was assembly with the integration plasmid pCfB2055 using Gibson Assembly^58^ and digested with NotI prior to integration. Colony-PCR using OneTaq (Thermo Scientific, Waltham, MA, USA) was used for confirming the integration of pCfB2055_GFP. Primers flanking the integration was used to confirm the integration. One single amplification band on gel electrophoresis indicated a successful integration into both chromosome copies. Genomic DNA extraction was performed as described previously^59^.

To generate the uracil knockout strain, the gBlock with *URA3* gRNA^34^ was assembled with pCfB2312 (Cas9-KanMX) to build the pCfB2312_URA and transformed together with URA-donor-fw primer (TCCATGGAGGGCACAGTTAAGCCGCTAAAGGCATTATAAGCCAAGTACAATTTTTTACTC) and URA-donor-rv primer (ACCAATGTCAGCAAATTTTCTGTCTTCGAAGAGTAAAAAATTGTACTTGGCTTATAATGC).

*S. boulardii* transformations were performed via high-efficiency yeast transformation using the LiAc/SS carrier DNA/PEG method^60^ with a minor modification of the incubation and heat-shock temperatures of 37 and 45 °C were used, respectively.

### Real-time growth monitoring

Real-time OD600 measurements were obtained every 5 min for approximately 24 h with microplate reader Synergy™ H1 BioTek. The cultures were incubated into 200 µL YPD in a CELLSTAR® 96 well cell culture plate (Greiner Bio-One) with an air-penetrable lid (Breathe-Easy, Diversified Biotech) supplemented with 0.3 g/L ampicillin sodium salt, 0.3 g/L kanamycinsulfate (Sigma Aldrich—Merck Life Science), 0.3 g/L metronidazole (Sigma Aldrich) and 0.15 g/L vancomycin hydrochloride (MP Biomedicals, LLC). Cultivation was performed with continuous double orbital shaking of 548 cycles per minute (CPM) at 37 °C and with an initial OD600 of 0.10.

### Animal study

All animal experiments were conducted according to the Danish guidelines for experimental animal welfare and the study protocols were approved by the Danish Animal Experiment Inspectorate (license number 2020-15-0201-00405). The study was carried out in accordance with the ARRIVE guidelines^61^. Unless otherwise stated, all mice were housed on a 12-h light/dark cycle and given ad libitum access to standard chow (Safe Diets, A30) and water. For the high fat diet studies, 45% high fat diet (Research Diet, D12451 Rodent Diet with 45 kcal% fat) was fed ad libitum*.* Mouse strains used in this study were female NMRI mice (6–8 weeks old; Taconic Bioscience), male *ob/ob* mice – B6.Cg-Lep^ob^/ J (The Jackson Laboratory) and male C57BL/6 mice (6–8 weeks old; Taconic Bioscience). All mice were randomised according to body weight and were acclimated for at least one week prior to the first oral administration of *S. boulardii*. The cohorts received the antibiotic cocktail (0.3 g/L ampicillin sodium salt, 0.3 g/L kanamycinsulfate, 0.3 g/L metronidazole, and 0.15 g/L vancomycin hydrochloride) supplemented in the drinking water, for up to five days prior to the first oral administration of *S. boulardii*. A single batch of *S. boulardii* was cultivated for 72 h before being harvested and frozen. The batch was used for the entire study. The impact of preservation of *S. boulardii* at − 80 °C in cryovials over time was assessed. Viable *S. boulardii* was assessed three, five and ten days after being frozen. Colony forming unit (CFUs) were count two days after plating (Supplementary Fig. S2). Each animal study received a freshly prepared batch of *S. boulardii*. The researcher was unblinded for all mouse experiments.

### Assessing the residence time (Fig. 1)

The NRMI mice were divided into two cohorts (n = 16), with and without antibiotics supplemented to the drinking water. Both cohorts were orally administered via intragastric gavage with ~ 10^7^ CFU of *S. boulardii* in 100 µL of 1 × PBS and 10% glycerol. The mice were orally administered *S. boulardii* for five consecutive days and faeces were collected daily for 20 days. On day five, half of the mice from each cohort (n = 8) were sacrificed. The gut content from six gastrointestinal tract regions was collected in 1 mL of 1 × PBS and 50% glycerol and weighed to determine the viability per gram of content.

### Assessing the effect of antibiotics (Fig. 2)

The NRMI mice were divided into two cohorts (n = 6), one with and one without antibiotic-treatment. Both cohorts were orally administered via intragastric gavage with ~ 10^8^ CFU of *S. boulardii* in 100 µL of 1 × PBS and 10% glycerol. The mice were orally administered *S. boulardii* for five successive days and faeces were collected daily. The cohort receiving the antibiotic cocktail in drinking water also received an oral administration of antibiotics (1.0 g/L ampicillin sodium salt, 1.0 g/L kanamycinsulfate, 1.0 g/L metronidazole, and 1.0 g/L vancomycin hydrochloride).

### Assessing the robustness of the antibiotic cocktail (Fig. 3)

The C57BL/6 mice (n = 9) and *ob/ob* mice (n = 9) were orally administered via intragastric gavage with ~ 10^8^ CFU of S. boulardii *URA3*∆ background strain in 100 µL of 1 × PBS and 10% glycerol to test the robustness of a strain without an antibiotic selection marker. The mice were housed individually to reduce the shared gut microbiota composition between each mouse^62^. In addition to receiving the antibiotic cocktail in drinking water, the mice also received an oral administration of the antibiotic cocktail (1.0 g/L ampicillin sodium salt, 1.0 g/L kanamycinsulfate, 1.0 g/L metronidazole, and 1.0 g/L vancomycin hydrochloride).

### CFU assessment in the faecal samples

The faeces were collected in pre-weighted 1.5 mL Eppendorf tubes containing 1 mL of 1 × PBS and 50% glycerol and weighed again to determine the faecal weight. All sample preparation for assessing CFU numbers was kept at 4 °C and followed the same practice. The faecal samples were homogenised and vortexed at ~ 2400 rpm for 10 min. The samples were then spun down at 100 g for 30 s, followed by a dilution series, where 5 µL of each dilution was plated in duplicates or triplicates. The faecal samples were plated on YPD plates containing 200 mg/L G418 for selecting the *S. boulardii* containing the KanMX marker. For selecting the uracil knockout *S. boulardii* the faecal samples were plated on SC plates containing 100 mg/L ampicillin, 50 mg/L kanamycin, and 30 mg/L chloramphenicol (Sigma Aldrich—Merck Life Science) for preventing bacterial growth and 1 g/L 5-Fluoroorotic Acid (5-FOA; Nordic BioSite) for preventing other yeast species to grow. To lower the limit of detection, 100 µL of the faecal samples was plated in duplicates from day 10 (Fig. 1b).

### Real-time quantitative PCR

According to the manufacturer's instructions, all genomic DNA were extracted with the DNeasey PowerSoil Pro kit (Qiagen, Hilden, Germany). The DNA concentration was determined with Qubit (Qubit™ dsDNA HS Assay Kit, Thermo Scientific, Waltham, MA, USA). 2 × SensiFAST SYBR® Lo-ROX mix (Nordic BioSite) was used for all qPCR reactions, consisting of 5 µL of master mix, 0.03 µL of each primer (100 µM, see Supplementary Table 1) and 5 µL of 0.1 ng/L of extracted template DNA. All qPCR reactions followed the same thermocycler program that consisted of an initial 3 min step at 95 °C, followed by 40 cycles of 95 °C for 10 s, 60 °C for 15 s, and 72 °C for 30 s, along with a final melting curve consisting of a single cycle of 95 °C for 5 s, 60 °C for 1 min, 95 °C for 5 s. All samples were run in duplicate.

Bacterial cell counts were determined using 16S rRNA and GFP specific primers for the total bacteria and *S. boulardii* cell counts, respectively (Supplemented Table S1). Cell counts were calculated based on a standard curve generated using a series of OD from either *E. coli* or *S. boulardii* and yielded genomic DNA concentration. We adjusted the qPCR signal of 16S and GFP by dividing them by the mean copy numbers per genome^63^; this was done to account for the fact that the 16S and GFP copy numbers per genome vary between species^63–65^, which consequently skew the readout of bacterial and *S. boulardii* cell counts. qPCR runs were performed on the Roche LightCycler® 480 Real-time PCR System in LightCycle® 480 Multiwell Plate 384, clear plates.

### Statistical testing

All statistical analysing were performed in RStudio. The normality of the data was checked with the Shapiro–Wilk test. A dependent sample t-test was used for normally distributed data and the Wilcoxon singed-rank test for non-normal distributed data.* P* values < 0.05 were considered statistically significant.

## Supplementary Information

Supplementary Information.

## Data Availability

All data generated or analysed during this study are included in this published article and its Supplementary Information files.
