# Supplementary material for: Effects of broad-spectrum antibiotics on the colonisation of probiotic yeast Saccharomyces boulardii in the murine gastrointestinal tract
Source: Sci Rep. 2022 May 25;12:8862. doi: 10.1038/s41598-022-12806-0 (PMC9133042; doi:10.1038/s41598-022-12806-0)
Supplement: Supplementary file 1 — Supplementary Information. [file 41598_2022_12806_MOESM1_ESM.pdf]

Supplemental Material for:

Effects of broad-spectrum antibiotics on the colonisation of probiotic yeast *Saccharomyces boulardii* in the murine gastrointestinal tract

Karl Alex Hedin[1], Vanessa Emily Rees[1], Hongbin Zhang[1], Vibeke Kruse[1], Ruben Vazquez-Uribe[1,*], Morten Otto Alexander Sommer[1,*]

1. Novo Nordisk Foundation Center for Biosustainability, Technical University of Denmark, DK2800 Kgs. Lyngby, Denmark

*Corresponding authors
Morten O.A. Sommer (msom@bio.dtu.dk)
Ruben Vazquez-Uribe (ruvas@biosustain.dtu.dk)

Supplementary Figures

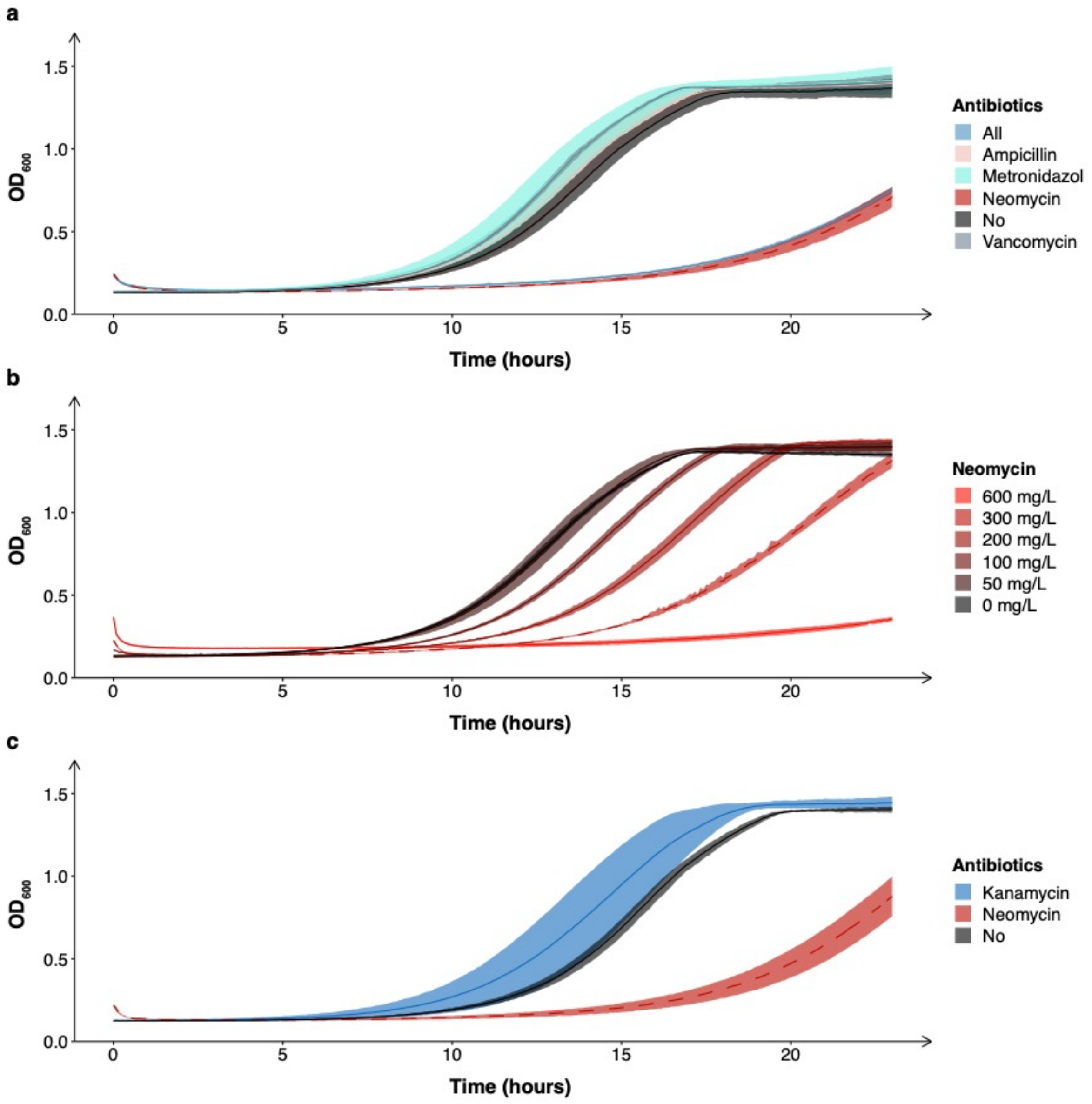

Figure S1. Growth performance on the antibiotics used in the antibiotic cocktail. (**a**) Individually and combined growth assessment on 300 mg/L ampicillin, 300 mg/L metronidazole, 300 mg/L neomycin and 150 mg/L vancomycin. (**b**) Growth assessment on a gradient of neomycin concentration. (**c**) Growth assessment on 300 mg/L kanamycin. Mean OD was obtained from four biological replicates ± SD monitored with microplate reader Synergy^{TM} H1 BioTek.

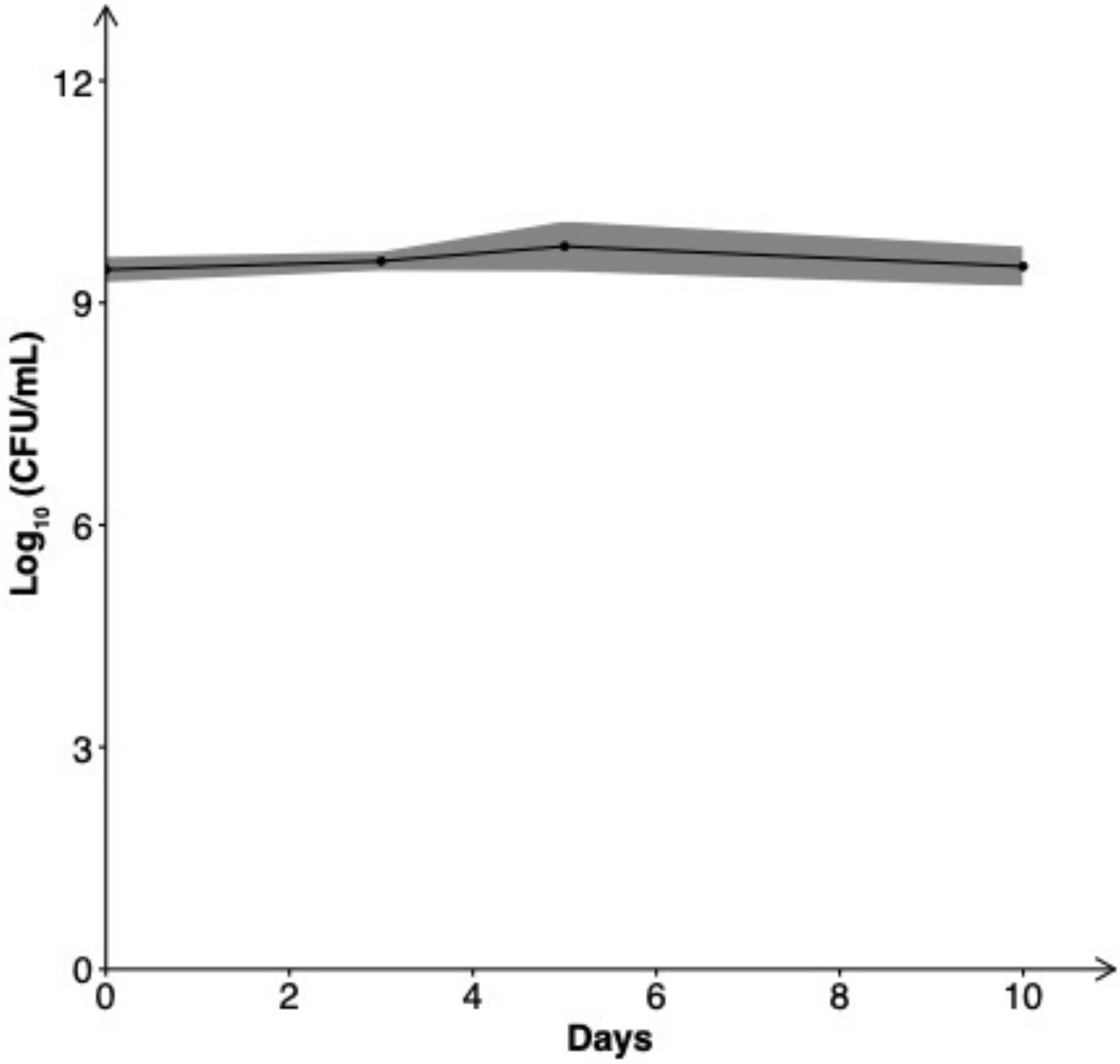

Figure S2. CFU assessment of *S. boulardii* at -80 °C over time. Line plot of the counted CFU per mL of *S. boulardii* in the cryovials for day 0, 3, 5 and 10.

Supplementary Tables

Table S1. Oligonucleotides used in this study

**Oligo Name**	**Sequence**	**Reference(s)**
p2055_GFP_fw	AGACAACCUGAAGTCTAGGTCCCTATTTATTTT	This study
p2055_GFP_rv	ATTTTGGAAUGCACGCGATCGCACGCAT	This study
TEF1p_GFP_fw	ATTCCAAAAUGTTTCTACTCCTTTTTTA	This study
TEF1p_GFP_rv	ACATTTTGUAATTAAAACTTAGATTAGA	This study
GFP_fw	ACAAAATGUCCAAAGGTGAAGAACTTTT	This study
GFP_rv	AGGTTGTCUAACTCCTTCCTTTTCGGT	This study
X-2_dg_fw	ATCGCCACTGACCCCATATT	This study
X-2_dg_rv	GTCTACTTGTCATAACTCAATTTGCC	This study
16S-V7-FW	GTGGTGCACGGCTGTCGTCA	[1]
16S-V7-RV	ACGTCATCCACACCTTCCTC	
qPCR_GFP_fw	TCTGTATCAGGTGAAGGAGAGG	This study
qPCR_GFP_rv	GGCATAGCAGACTTAAAGAAG	This study

Table S2. Strains used in this study

**Strain**	**Genotype**	**Marker**	**Parental strain**	**Reference**
SB-ATCC-796	WT	N/A	NA	This study
Sb KanMX:GFP (X-2)	p2055 KanMX:GFP	KanMX	SB-ATCC-796	This study
SbU	*URA3Δ*	N/A	SB-ATCC-796	This study

Table S3. Plasmids used in this study

**Plasmid name**	**Genotype**	**Marker (E.coli / S. cerevisiae)**	**Reference**
pCfB353	X-2-loxP-KanMX	Amp / kanMX	[2]
pCfB2055	pCfB2055	Amp / kanMX	pCfb353 derivative

pCfB2055-GFP	pCfB2055; P_{TEF1}-GFP-T_{CYC1} kanMX	Amp / kanMX	This study
pCfB2312	CEN6_ARS4 Amp P_{TEF1}-Cas9-T_{CYC1} kanMX	Amp / kanMX	[3]
pCfB2312 - URA3	pCfB2312; gRNA-*URA3*	Amp / kanMX	This study

REFERENCE

[1] Guillen, I. A. *et al.* PCR Conditions for 16S Primers for Analysis of Microbes in the Colon of Rats. (2016) doi:10.7171/jbt.16-2703-002.

[2] Jensen, N. B. *et al.* EasyClone: method for iterative chromosomal integration of multiple genes Saccharomyces cerevisiae. *FEMS Yeast Research* **14**, 238–248 (2014).

[3] Jessop-Fabre, M. M. *et al.* EasyClone-MarkerFree: A vector toolkit for marker-less integration of genes into Saccharomyces cerevisiae via CRISPR-Cas9. *Biotechnology Journal* **11**, 1110–1117 (2016).
